# Short Peptide with Sequence of LAGAAHF, Identified from Edible Bird’s Nest, Reduces Dermatitis Symptoms in Mice

**DOI:** 10.3390/ph19040649

**Published:** 2026-04-21

**Authors:** Queenie Wing Sze Lai, Yaxin Wang, Shengying Lin, Gary Ka Wing Yuen, Dusadee Ospondpant, Alex Xiong Gao, Tina Ting Xia Dong, Xuncai Liu, Qunyan Fan, Karl Wah Keung Tsim

**Affiliations:** 1 Shenzhen Key Laboratory of Edible and Medicinal Bioresources, Shenzhen Research Institute, The Hong Kong University of Science and Technology, Shenzhen 518057, China; 2 Division of Life Science and Center for Chinese Medicine R&D, The Hong Kong University of Science and Technology, Clear Water Bay, Kowloon, Hong Kong SAR, China; 3 Bird’s Nest Research Institute, Xiamen Yan Palace Seelong Biotechnology Co., Ltd., Xiamen 361100, China

**Keywords:** edible bird’s nest, TNF-α signaling pathway, reactive oxygen species, NF-κB signaling pathway, filaggrin

## Abstract

**Background/Objectives**: Native to the Indo-Pacific region, edible bird’s nests (EBN; Yan Wo in Chinese) are the solidified saliva of swiftlets (*Aerodramus fuciphagus* and *A. maximus*) and have been consumed as a traditional functional food for centuries. However, the bioactive components and underlying mechanisms of EBN remain poorly understood. EBN consists of over 60% protein, much of which is heavily glycosylated, forming complex glycoconjugates that are resistant to enzymatic digestion. This study examines the properties of EBN-derived bioactive peptides and assesses their potential for skin moisturization and anti-inflammation when applied topically. **Methods**: EBN was double-boiled for an extended period, then digested with gastric enzymes to extract active peptides. Digestion was over 90% efficient, and peptide molecular weights were measured. The enzymatic digest was then fractionated using an activity-guided approach based on assays for skin moisturization and anti-inflammatory properties. **Results**: A novel bioactive heptapeptide, with the sequence LAGAAHF and designated EBN_P3_, was identified and characterized. It attenuated TNF-α-induced inflammatory responses in HaCaT keratinocytes and alleviated dermatitis symptoms in a DNCB-induced C57BL/6 mouse model. **Conclusions**: EBN-derived peptides with skin moisturizing and anti-inflammatory activities hold significant promise for development into functional ingredients for skincare products.

## 1. Introduction

Active ingredients in skincare products were originally based on petroleum as a humectant to retain skin moisture. Today, consumers begin to pursue cosmetics that have active ingredients from natural products, focusing on discovering the functions of small molecules, e.g., retinol and salicylic acid. Nevertheless, small molecules may possess adverse effects, e.g., irritation and carcinogenicity, which arouse public concerns about the safety and efficacy of cosmetic products [1]. Thus, peptides derived from natural products with skin-enhancing properties are considered excellent ingredients for skincare products. In general, cosmetic peptides exhibit skin-protective activities against allergies, microbes, hyperpigmentation, and oxidative damage; additionally, these peptides are mostly classified as inhibitors for neurotransmitters, enzymes, and carrier proteins [2,3,4,5].

Edible bird’s nests (EBN, Yànwō) are made from the saliva of swiftlets, mainly from *Aerodramus fuciphagus* and *A. maximus*, and consist > 60% protein [6]. EBN is well known to promote skin functions, e.g., improving skin complexion [7], reducing signs of aging [8], promoting wound-healing [8], restoring skin lightness [9], facilitating skin moisturization [10], and inhibiting inflammation [10]. Indeed, the digested EBN peptides have been shown to have moisturizing and anti-inflammatory properties in skin. The enzymatic digestion of EBN liberates N-acetylneuraminic acid (NANA) in its free form along with low-molecular-weight peptides, thereby enhancing the targeted skin functions of EBN. EBN extract and digest comprised approxiamately 4.32% and 11.43% (*w*/*w*) NANA, respectively. The free NANA in EBN does not fully explain EBN’s skin functions [10]. Peptides from enzymatic digestion likely contribute to skin moisturizing and anti-inflammatory effects. Although EBN has traditionally been consumed as a nutritional supplement, emerging evidence indicates that food-derived bioactive peptides can exert systemic effects beyond nutrition, including modulation of inflammation, oxidative stress, and skin health. Accordingly, this study aims to identify bioactive peptides from EBN and evaluate their skin-moisturizing and anti-inflammatory properties, bridging traditional use with modern translational applications.

The bottleneck in the proteomics study of EBN is a high density of hardly digested glycoprotein and a lack of a complete genomic sequence of *Aerodramus* [11]. Despite progress in de novo sequencing, a comprehensive EBN protein profile is still missing. Here, double boiling and simulated gastrointestinal digestion were performed under physiologically relevant conditions to mimic traditional preparation and human digestion. Protein denaturation during these processes facilitates the release of bioactive peptides from glycoprotein precursors. Small peptides isolated from the digested EBN demonstrated significant induction of filaggrin and filaggrin-2 expressions, along with suppression of inflammatory responses, in cultured skin keratinocytes. The identified EBN peptides were further validated in an animal model of skin dermatitis. These EBN peptides offer improved bioavailability, enhanced skin absorption, reduced toxicity, and limited allergenic risk for cosmetic use [12].

## 2. Results

### 2.1. Identification of Active Peptides

The EBN hydrolysate, obtained after over-stewing and simulated gastric digestion, was characterized by HPLC with a size-exclusion column. Finding anti-inflammatory bioactive small peptides was the target. Low-molecular-weight peptides below 3 kDa were isolated via reverse-phase chromatography through an activity-guided fractionation strategy. These peptide fractions were labeled with TMT isobaric mass tags for analysis by Orbitrap Eclipse. Peptide features were consistently detected within defined *m*/*z* ranges and charge states (+2 to +4), with identifications filtered at FDR < 1%. MS/MS spectra exhibited clear b- and y-ion fragmentation patterns, and peptide features (*m*/*z* and retention time) were reproducible across TMT channels and replicates, supporting the preservation of peptide integrity during fractionation.

The resulting MS data were then searched by PEAKS Studio against 3 Uniprot databases: *Apodidae*, *Chaetura pelagica*, and *Aerodramus*. The sequences were matched with proteins from *Apodidae* and *C. pelagica* databases. Amongst the identified peptides, one peptide, named EBN_P3_, was identified showing robust activities in skin functions. The identified peptide was subjected to protein sequence analysis by LC–MS/MS. LC–MS/MS analysis of the bioactive fraction identified multiple low-molecular-weight peptides within the *m*/*z* range of 350.2128–991.7618. By comparing to data banks from *Apodidae* and *C. pelagica*, the sequence identity of EBN_P3_ was revealed as LAG AAH F, having 7 amino acids. The target heptapeptide was consistently detected with high signal intensity. The extended characterized EBN_P3_ was proposed to derive from pigment epithelium-derived factor with a proposed pI at 7.89 with zero charge. EBN_P3_ did not alter cell viability in cultured HaCaT keratinocytes up to 0.5 mg/mL (Supplementary Materials, Figure S1A). Thus, EBN_P3_ was chemically synthesized and evaluated in subsequent functional assays to validate the bioactivity.

**Figure 1:**
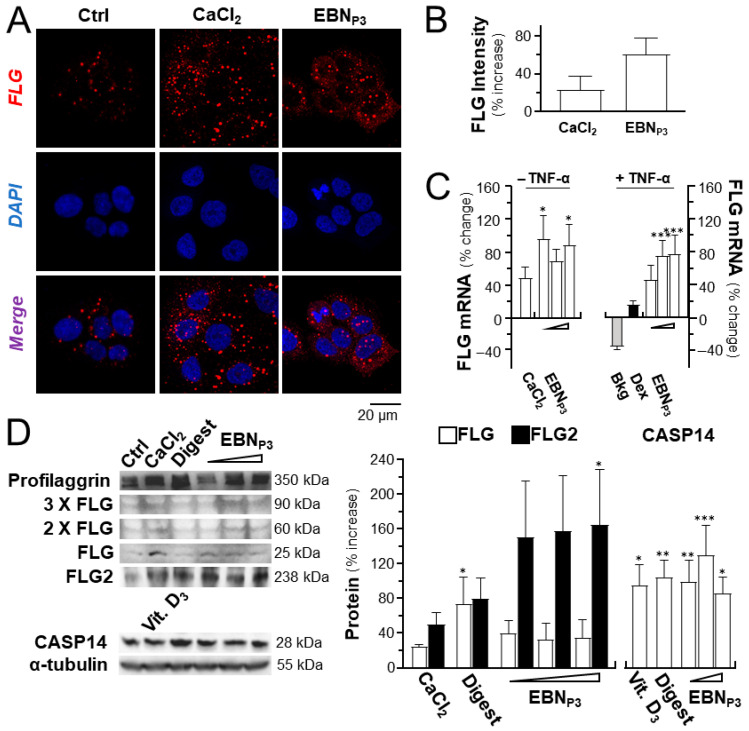
EBN_P3_ induces the expression of moisturizing markers. (**A**) The confocal microscopic imaging of filaggrin proteins was visualized in HaCaT keratinocytes after 24 h incubation with EBN_P3_ at 1 μg/mL. CaCl_2_ (0.16 mM) served as a positive control. (**B**) The fluorescence intensity was quantified. (**C**) EBN_P3_ was applied onto cultured HaCaT cells with or without TNF-α at 20 ng/mL. The mRNA expressions of filaggrin (FLG) after 24 h treatments of EBN_P3_ (at 0.1, 1, and 10 μg/mL) and CaCl_2_ (at 0.16 mM) were measured. (**D**) Representative Western Blot membranes show the protein bands of filaggrin (FLG) at different oligomers, filaggrin-2 (FLG2), and CASP14 (**left panel**). The protein expressions in HaCaT keratinocytes were assessed after 24 h of treatment with EBN_P3_ (0.1, 1, 10 μg/mL). Positive controls were CaCl_2_ (at 0.16 mM), cholecalciferol (vitamin D3, at 10^−5^ M), and EBN digest (at 100 μg/mL). The relative level of the proteins was quantified, normalized to α-tubulin (right panel). Values are reported as a percentage or a fold change relative to the basal. A statistical difference relative to the control or the TNF-α-treated group was analyzed by one-way ANOVA and reported herein as mean ± SEM, * *p* < 0.05, ** *p* < 0.01, and *** *p* < 0.001, *n* = 4.

In the cultured keratinocytes, the application of EBN_P3_ induced the expression of filaggrin, a biomarker for skin moisturization. By immunohistochemical analysis, the immunofluorescence intensity of filaggrin in the treated cultures was revealed, which showed an increase of ~60% under the treatment of the peptide (Figure 1A,B). CaCl_2_ was a positive control. In parallel, the mRNA induction was identified under EBN_P3_ treatment with or without TNF-α (Figure 1C). The EBN peptide showed better results than the CaCl_2_ control. The protein levels of filaggrin, filaggrin-2, and the downstream CASP14 protease for the generation of natural moisturizing factors were assessed by Western Blotting in the EBN_P3_-treated cultures: the treatment significantly induced the expression of total filaggrin (including profilaggrin) and filaggrin-2, as well as CASP14 (Figure 1D).

**Figure 2:**
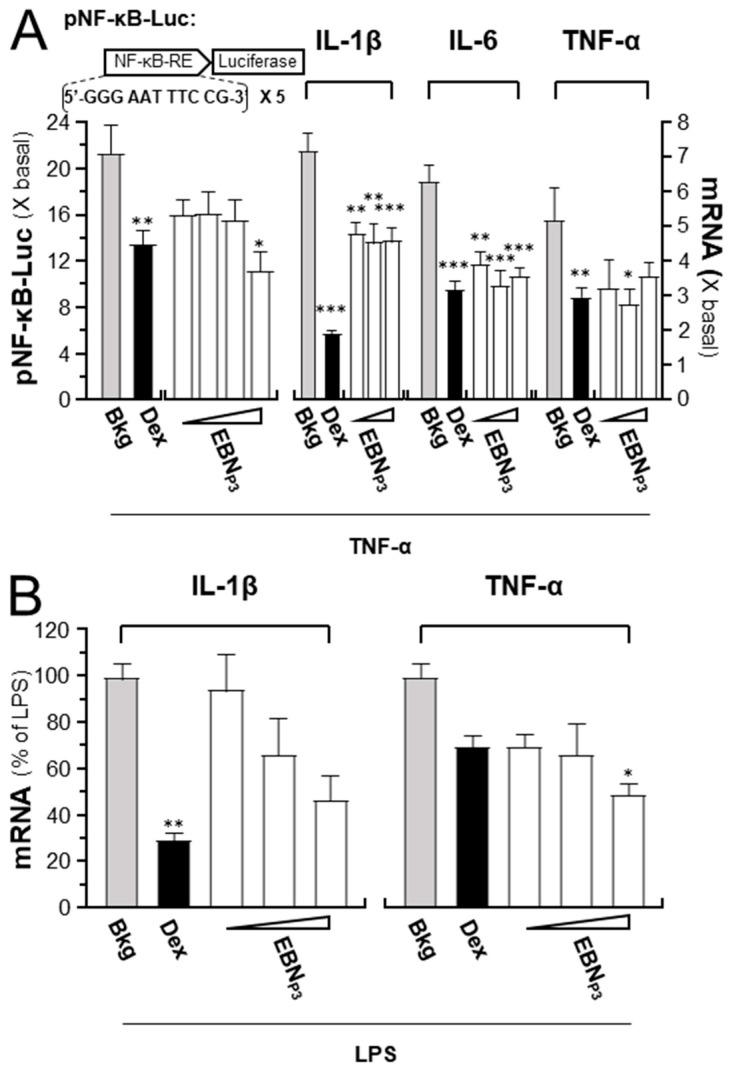
EBN_P3_ suppresses TNF-α-mediated inflammatory responses. (**A**) Cultured HaCaT cells were transfected with the pNF-kB-Luc DNA construct (left panel). The NF-κB-driven luciferase activity after 2 h incubations of TNF-α (20 ng/mL) with EBN_P3_ (at 0.01, 0.1, 1, and 10 μg/mL) was measured. The mRNA levels of IL-1β, IL-6, and TNF-α, after 2 h incubations of TNF-α with EBN_P3_ (at 0.1, 1, and 10 μg/mL), and dexamethasone (at 10 nM, positive control), respectively. (**B**) The levels of IL-1β and TNF-α mRNA in RAW264.7 cells, following a 3 h incubation with EBN_P3_ (at 0.1, 1, and 10 µg/mL) and a subsequent 24 h LPS (at 100 ng/mL) activation. Dexamethasone (at 100 µM) was used as a positive control. Results are presented as fold changes relative to baseline or the percentage of LPS activation. A statistical difference compared with the TNF-α- or LPS-treated group was analyzed by one-way ANOVA and expressed as mean ± SEM. Significance: * *p* < 0.05, ** *p* < 0.01, and *** *p* < 0.001, *n* = 4.

To reveal the anti-inflammatory effects of EBN_P3_, cultured HaCaT cells were transfected with the pNF-κB-Luc reporter DNA construct. After DNA transfection, luciferase activity driven by the reporter increased ~22-fold at 2 h of TNF-α stimulation, while simultaneous treatment with high doses of EBN_P3_ markedly suppressed ~60% of the activated activity (Figure 2A). In addition, exposure to TNF-α induced a >5-fold increase in the mRNA levels of the pro-inflammatory cytokines IL-1β, IL-6, and TNF-α: this induction was inhibited significantly by co-incubation of the EBN_P3_ peptide at different doses (Figure 2A). In cultured RAW264.7 cells, EBN_P3_ treatment resulted in dose-dependent suppression of LPS-induced IL-1β and TNF-α mRNA, with inhibition rates of 5.5–53% and 30–50.5%, respectively (Figure 2B). Dexamethasone was a positive control. The results suggested that the identified EBN peptide was able to promote skin hydration and reduce inflammation by facilitating the expression of specific biomarkers in skin keratinocytes.

### 2.2. Functional Exploration of EBN_P3_

**Figure 3:**
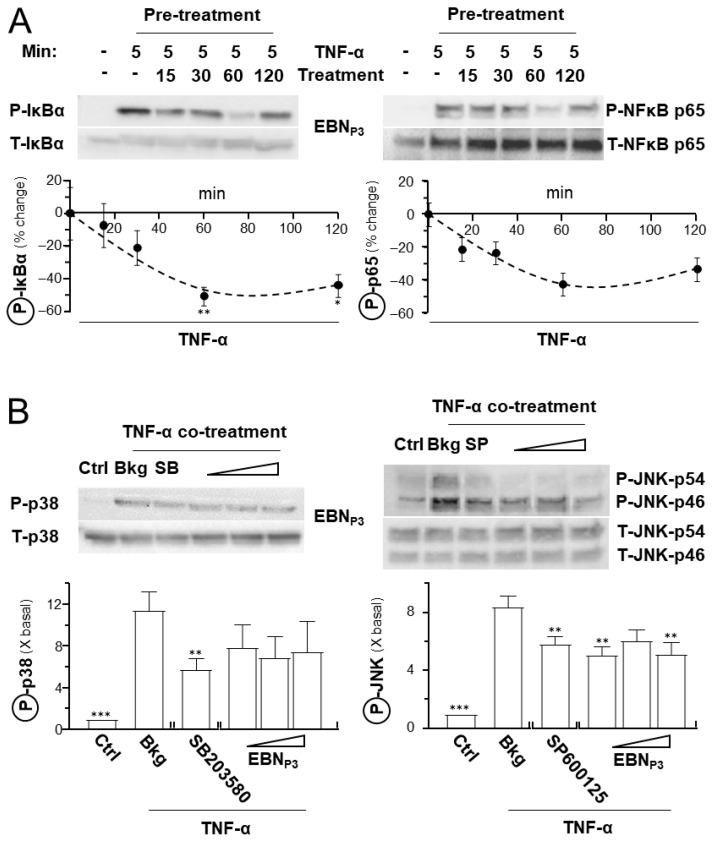
EBN_P3_ reduces the TNF-α-induced Iκ-Bα, NF-κB p65, and MAPK phosphorylations. (**A**) Representative Western Blot membranes show the phosphorylated and total Iκ-Bα and NF-κB p65 (upper panel). The 16 h starved cultures were pretreated without or with EBN_P3_ (at 10 μg/mL) for 15–120 min before 5 min TNF-α (at 20 ng/mL) stimulation. The lower panel shows the quantification. (**B**) Representative blots showing phosphorylated and total p38 and JNK protein bands (upper panel). The 16 h starved cultures were simultaneously incubated for 20 min with TNF-α (at 20 ng/mL) and EBN_P3_ (at 0.1, 1, 10 μg/mL). SB203580 (at 10 µM) and SP600125 (at 10 nM) served as positive controls, respectively. The relative phosphorylation levels were quantified against basal after normalizing the band intensities of phosphorylated proteins with those of total proteins (lower panel). Results are presented as the fold changes relative to baseline, or the percentage of LPS activation. Values are expressed as the percentage change to the TNF-α-treated group, or as the fold change in basal. A statistical difference compared with the TNF-α-treated group was analyzed by one-way ANOVA and expressed as mean ± SEM. Significance: * *p* < 0.05, ** *p* < 0.01, and *** *p* < 0.001, *n* = 3.

**Figure 4:**
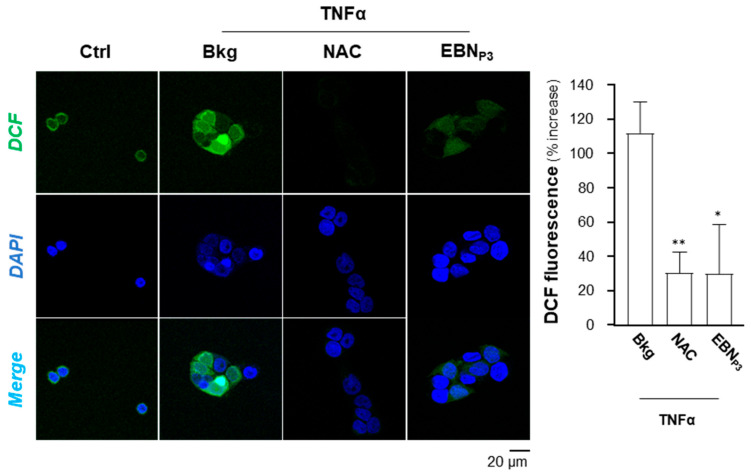
EBN_P3_ inhibits TNF-α-induced intracellular ROS. Representative confocal microscopy images showing the intracellular ROS indicator, DCF. HaCaT keratinocytes were pre-treated for 2 h with EBN_P3_ (10 μg/mL), followed by 5 min of TNF-α stimulation. Intracellular ROS was then stained by a 20 min incubation with DCFH-DA (**left panel**). NAC (at 20 μM) was used as a positive control. DCF fluorescence (Alexa Fluor^®^ 488, green) was normalized to cell number (**right panel**) and expressed as a percent increase relative to the control (set at 0). Nuclei were counterstained with DAPI (blue). Colocalization of DCF and DAPI is shown in the merged panels. A statistical difference compared with the TNF-α-treated group was analyzed by one-way ANOVA and expressed as mean ± SEM. Significance: * *p* < 0.05, ** *p* < 0.01, *n* = 3.

The potential of EBN_P3_ to suppress NF-κB signaling in HaCaT cells was assessed by determining its effects on key inflammatory mediators, e.g., Iκ-Bα, NF-κB p65, and p38. The TNF-α-activated Iκ-Bα and NF-κB p65 phosphorylations were markedly suppressed by EBN_P3_ over time (Figure 3A). Additionally, p38 and JNK phosphorylations were both induced by application of TNF-α in cultured HaCaT cells and suppressed by EBN_P3_ (Figure 3B). SB203580, a p38 MAPK inhibitor, served as a control. In addition, the potential of EBN_P3_ in inhibiting the TNF-α-induced intracellular ROS level was evaluated. The fluorogenic dye, DCFH-DA, was employed to indicate the level of ROS in cultured keratinocytes. HaCaT cells were incubated for 2 h with EBN_P3_, prior to a 5 min stimulation by TNF-α (at 20 ng/mL). NAC (at 20 μM) served as a positive control. The DCF green fluorescence, induced by the applied TNF-α, was markedly reduced by more than 60% in the presence of EBN_P3_, as well as the positive control (Figure 4).

**Figure 5:**
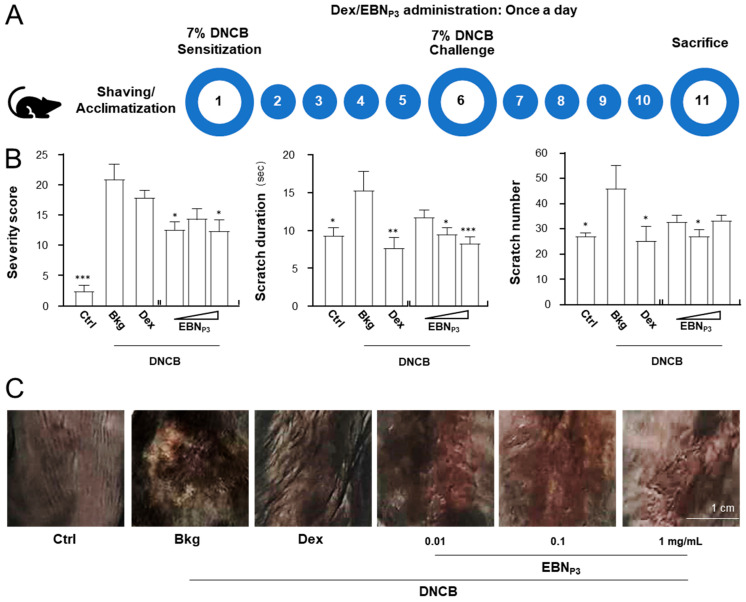
EBN_P3_ ameliorates the behavioral, biological, and immunological characteristics in DNCB-induced AD mice. (**A**) The 11-day experimental procedure of the DNCB-induced C57BL/6 model. Mice were acclimatized, shaved, and initially sensitized with 7% (*w*/*w*) DNCB on dorsal skin. EBN_P3_ (at 0.01, 0.1, 1 mg/mL) was applied daily. DNCB sensitization was repeated on day 6, and mice were euthanized on day 11. Mice treated with vehicle and dexamethasone (0.12% *w*/*w*) were used as the control and positive control groups. (**B**) The severity of dermatitis symptoms and scratching duration and frequency were assessed daily. A simplified SCORAD scoring system was used for visual assessment of dermatitic skin characteristics: each criterion was scored as 1 point for its presence (rather than intensity) to minimize subjective variation. Scratching frequency (number of bouts) and duration (seconds) were measured daily over a 30-min observation period. Values are expressed in absolute time and number. A statistical difference compared with the DNCB-treated group was analyzed by one-way ANOVA and expressed as mean ± SEM. Significance: * *p* < 0.05, ** *p* < 0.01, and *** *p* < 0.001, *n* = 4. (**C**) Representative images of mouse dorsal skin, after each treatment, are shown.

Prior to functional skin testing in animals, 2-week dorsal application of EBN_P3_ (single dose at 1000 mg/kg) induced no acute dermal toxicity in mice [13,14,15]. Comparable skin thickness was observed in both control and treated groups, with no signs of erythema, edema, atonia, desquamation, fissuring, scab formation, exfoliation, or tissue damage/necrosis, as shown in Supplementary Materials (Figure S1B). The animal model of dermatitis was used to determine the efficacy of EBN_P3_. The DNCB-induced mice were employed to examine the effects of EBN_P3_. The experimental procedure was depicted in Figure 5A. The skin-relieving properties of EBN_P3_ were assessed during an 11-day topical treatment along with the dorsal skin sensitizations on days 1 and 6. Dexamethasone (at 0.012% *w*/*w*) and EBN digest (at 1 mg/mL), prepared in a cream, were adopted as the controls. The presence of signs of skin inflammation, i.e., erythema, edema/papulation, oozing/crusts, excoriations, lichenification, dryness, vesicles, and pigmentation/depigmentation, was observed following the DNCB treatment. The visual assessment of dermatitic skin characteristics was analyzed with the simplified version of SCORAD scoring [16]. The DNCB-induced severity score was found to be significantly reduced in the presence of EBN_P3_ (Figure 5B). The scratch frequency and duration within a daily 30 min observation were shown to be markedly reduced by 50% with the treatment of EBN_P3_ (Figure 5B). The pathological markers of the appearance of skin dermatitis were measured by histochemical analysis. The skin showed serious damage under the induced inflammation, which was much restored in the presence of EBN_P3_ and dexamethasone (Figure 5C).

**Figure 6:**
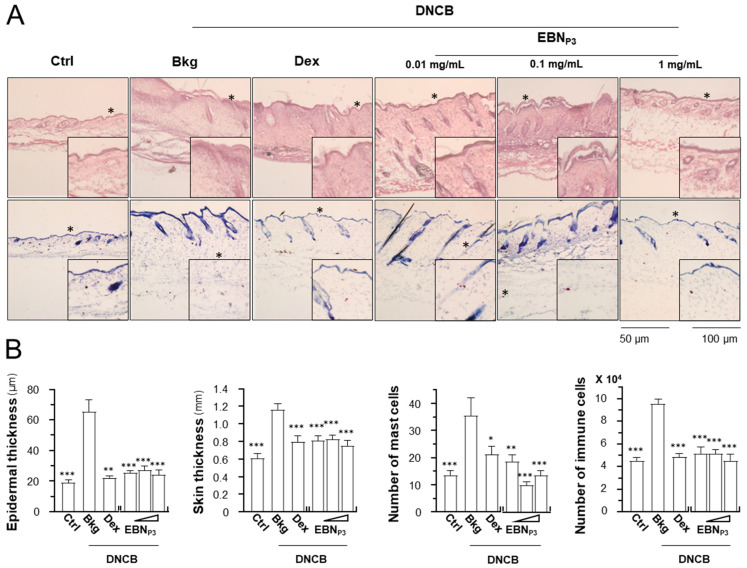
EBN_P3_ improves the skin characteristics of DNCB-induced AD mice. (**A**) The skin sections were obtained from the treated mice. The epidermal and skin thicknesses under each treatment were measured from H&E-stained sections at 5 randomly selected sites under a 10× microscopic field. The total numbers of mast cells and immune cells were counted in toluidine blue- and H&E-stained sections, respectively, across 5 randomly selected areas per mm^2^ under 10× magnification. (**B**) The epidermal thickness, skin thickness, number of mast cells, and number of immune cells were counted from different treatments. A statistical difference compared with the DNCB-treated group was analyzed by one-way ANOVA and expressed as mean ± SEM. Significance: * *p* < 0.05, ** *p* < 0.01, and *** *p* < 0.001, *n* = 4.

**Figure 7:**
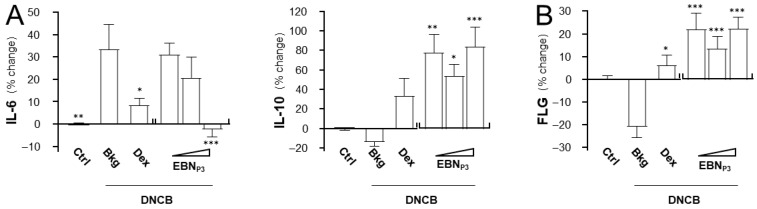
EBN_P3_ modulates skin inflammation and restores hydration biomarkers in DNCB-induced AD mice. The relative expression levels of (**A**) IL-6, IL-10, and (**B**) skin barrier protein filaggrin (FLG) were determined by ELISA assays in the excised dorsal skin. Values are standardized according to tissue protein content and reported as the percentage change relative to baseline. A statistical difference compared with the DNCB-treated group was analyzed by one-way ANOVA and expressed as mean ± SEM. Significance: * *p* < 0.05, ** *p* < 0.01, and *** *p* < 0.001, *n* = 4.

In the stained skin cryosections, the epidermal and skin thicknesses were measured, and populations of mast cells and immune cells were quantified. The DNCB-treated mice showed a robust increase in epidermal and skin thicknesses, as well as the numbers of mast cells and immune cells (Figure 6A,B). The treatment of EBN_P3_ suppressed the outcomes of DNCB-induced dermatitis markedly. Dexamethasone was a control. The levels of IL-6, IL-10, and the skin barrier protein filaggrin were quantified in dissected skin using ELISA. The expression level of IL-6 was robustly induced by ~30% while the level of IL-10 was inhibited by ~20% in the DNCB-treated mice (Figure 7A). The treatments of EBN_P3_ in all scenarios completely restored the impacts triggered by DNCB insult. In addition, the DNCB-suppressed filaggrin expression was markedly recovered by the treatment with EBN_P3_ (Figure 7B).

## 3. Discussion

EBN is a renowned health food supplement commonly consumed in Southeast Asia for its complexion-enhancing and anti-aging properties, and its use has been recorded for about 1400 years in China. EBN has various skin benefits, e.g., growth-stimulating effects on epidermal tissues, skin whitening, promoting wound healing, and reducing water loss, wrinkles, and dermal thickness. Although EBN has been included in cosmetic products, the active ingredients responsible for skin functions are still controversial. Uncertainties remain whether the cosmetic industries should focus on the hydrolysed product of EBN as a whole or on extracting the key components, i.e., peptides and NANA, from the complex proteins. The protein profiling of EBN has been studied and reported [17,18,19,20,21,22]; however, the exact protein identity remains unclear due to the adoption of different methods of protein hydrolysis and determination. Additionally, the lack of a complete genome of *Aerodramus* hinders the discovery of bioactive proteins and peptides derived from EBN [23]. Although various bioactive peptides have been searched from EBN hydrolysates with the advance of de novo sequencing, their identities are still very limited.

The skin moisturizing and anti-inflammatory assessments of EBN extract, digest, and NANA have suggested that the digest exhibits better functions [10,24]. Thus, the peptides and/or small compounds from the digested EBN could be the active ingredients [25]. The free NANA in EBN extract and digest were accounted for 4.32% and 11.43% (*w*/*w*) of the dried sample by LC–MS/MS analysis, respectively [24]. NANA serves as an authentication marker of EBN, where the reported concentrations align with previous findings [26]. By estimation, about 0.037 mM NANA was identified in EBN digest (at 100 μg/mL): NANA did not account for the full profile of anti-inflammatory responses at this level. Here, the findings support the proposed notion that small peptides could be the active ingredients of EBN. Additionally, the cutaneous benefits of EBN are well-documented and are believed to originate from its peptidomes [21,27,28], with hydrophobic tripeptides predominantly possessing wound-healing effects [21]. Despite the discovery of 2 pentapeptides, PFHPY and LLGDP from cytochrome b of EBN, which were reported for their antioxidative effects [29], no specific sequences have been identified for skin-protective effects. Although EBN has been previously reported to exhibit anti-inflammatory properties, these studies primarily relied on crude extracts or partially characterized fractions. In contrast, the present study identifies and validates a specific low-molecular-weight peptide associated with this activity. We believe this is the first report identifying a potential novel bioactive peptide sequence with skin moisturizing, anti-inflammatory, and antioxidative functions, supported by database comparison and synthetic peptide validation.

To isolate bioactive components from the complex edible bird’s nest (EBN) matrix, size-exclusion chromatography (SEC) and reverse-phase HPLC (RP-HPLC) were employed as complementary separation strategies based on molecular size and hydrophobicity. This orthogonal approach enabled enrichment of bioactive fractions, which were subsequently analyzed using TMT-based LC–MS/MS for multiplexed relative quantification and activity-guided prioritization of candidate peptides. While peptide identification was achieved through MS/MS fragmentation analysis, definitive structural confirmation may require additional orthogonal approaches to fully resolve protein identities and origins in EBN, which remain inconsistently reported in the literature.

Furthermore, although activity-guided fractionation enriched peptide-containing fractions, these fractions are unlikely to consist exclusively of peptides and may include co-eluting biomolecules such as glycans and other small components that contribute to the observed bioactivities. To address this, candidate peptides identified by LC–MS/MS were chemically synthesized and independently validated, confirming that specific peptide sequences contribute directly to the observed biological effects. This approach enables a focused evaluation of peptide-specific activity, independent of potential synergistic influences from other components present in the original fractions. Nevertheless, the bioactive fractions originate from a complex mixture of peptides and other biomolecules. Therefore, while the identified heptapeptide was validated as bioactive, the present findings support its role as a contributing factor rather than establishing a generalized effect for all EBN-derived peptides. Further studies are required to systematically evaluate additional peptides and their possible synergistic effects.

The short EBN peptide was proposed to originate from pigment epithelium-derived factor. The concentration of EBN_P3_ in the enzymatic digest was determined to be 3.21 ± 0.34% (*w*/*w*) using LC–MS/MS. Pigment epithelium-derived factor is a ~50 kDa secreted glycoprotein [30], which has been identified in human liver, kidney, heart, pigment epithelial cells, retinal epithelial cells, and adipocytes, as well as in human saliva [31], which is a member of the non-inhibitory subgroup of the serine protease inhibitor superfamily [2,30]. The functions of this protein have been proposed, including anti-inflammatory, antioxidative, anti-angiogenic, anti-thrombotic, as well as inhibition of tumor formation and support for brain health.

TNF-α-induced activation of NF-κB signaling is crucial for the pathogenesis of chronic skin inflammation [32]. Most pro-inflammatory effects mediated by TNF-α are triggered through the receptor signaling. Neutralizing the TNF-α-receptor signaling has shown beneficial effects in autoimmune and inflammatory conditions [33]. To evaluate the skin-protective anti-inflammatory activity of EBN-derived peptides, understanding their interactions with TNF-α is crucial, as these interactions could be the key to developing potential treatments for atopic dermatitis. As an initial attempt, computational docking was used to estimate the interaction between EBN_P3_ and TNF-α [34], assessing its potential to inhibit the interaction between TNF-α and its receptors. The docking score represents the top-ranked binding model of EBN_P3_ with TNF-α, where the ligand root-mean-square deviation (rmsd) measures how closely the predicted ligand position aligns with the input structure [35]. The most significant docking scores and values of shortest root-mean-square deviation for EBN_P3_ are compared with certolizumab’s light domain, a commercial TNF-α monoclonal antibody, used as a positive control.

The binding affinity of the EBN_P3_ peptide–TNF-α protein complex was evaluated through docking [36,37,38], which suggested potential inhibitory effects and therapeutic applications for skin inflammation. Molecular docking showed a possible binding of EBN_P3_ to TNF-α at an energy level, represented by HDOCK docking scores of −203.58 [39], compared to those of certolizumab (−256.90) (Supplementary Materials, Figure S1C). This binding could partly account for the functions of EBN_P3_ as described here; however, additional experimental work is needed for further confirmation.

## 4. Materials and Methods

### 4.1. Preparation of EBN Extract and Digest

The white EBN sample was obtained from Mountain & Sea Bird’s Nest Co. Limited (Hong Kong, China) and sourced from house-farmed *Aerodramus fuciphagus* in Malaysia. The samples were of standard “cup” grade. Authenticity was confirmed morphologically by professionals from the Hong Kong Chinese Medicine Merchants Association, with independent verification conducted by the Center for Chinese Medicine R&D and Xiamen Yan Palace Seelong Biotechnology Co., Ltd. to ensure consistency across multiple parties. Authenticity was further validated using molecular approaches, including reelin gene analysis. Sample consistency and quality were assessed through SEM–EDS, protein profiling, sialic acid quantification (≥10%, *w*/*w*), and total protein content analysis (≥60%). All raw samples were stored at 20–25 °C. To prepare the EBN extract, the raw material was first moisturized and expanded in 1:100 (*w*/*v*) double-deionized (DDI) water overnight. Water-soluble inorganics were then removed by rinsing the expanded EBN with DDI water 3 times. EBN was double-boiled in 1:30 (*w*/*v*) DDI water at 98 ± 2 °C under constant stirring for 8 h. The filtrate from the cooked EBN was collected, lyophilized, and designated as the EBN extract. To prepare the EBN digest, the EBN extract was subjected to complete digestion with 1:100 (*w*/*v*) simulated gastric fluid (SGF, catalog number: 01651) and 7.6% (*w*/*w*) pepsin (catalog number: P7012) (Sigma-Aldrich, St Louis, MO, USA) at 37 °C for 48 h. The digestion was terminated by 1:10 (*v*/*v*) 0.7 M NaCl solution. The neutralized product was lyophilized, designated as the EBN digest, and stored at −80 °C.

### 4.2. HPLC Separation and Fractionation

EBN digest was analyzed by HPLC-UV using an Agilent HPLC 1200 series system (Agilent, Waldbronn, Germany). EBN digest, prepared in DDI water, was subjected to fractionation by Superdex 75 gel filtration column (catalog number: GE17-5174-01) (GE Healthcare, Little Chalfont, UK). Peptides and proteins were detected at wavelengths of 214 and 280 nm, respectively [10]. Phosphate-buffered saline (PBS) mobile phase (containing 0.14 M NaCl, 2.68 mM KCl, 0.01 M Na_2_HPO_4,_ and 1.76 mM KH_2_PO_4_) at a flow of 0.4 mL/min was applied as eluent. A mixture of standard proteins comprising α-melanocyte-stimulating hormone (α-MSH, 1.7 kDa) (catalog number: M4135-1MG) (Sigma-Aldrich), aprotonin (6.5 kDa), ribonuclease A (13.7 kDa), carbonic anhydrase (29 kDa), ovalbumin (43 kDa), conalbumin (75 kDa), and blue dextran (>2000 kDa) (catalog number: 28-4038-41) (GE Healthcare) was employed for molecular weight calibration. Model 2110 fraction collector (Bio-Rad Laboratories, Hercules, CA, USA) was used to collect liquid eluted at 20–36, 36–52, 52–68, and 68–84 min. The fractionated eluents were lyophilized to prepare the digested fractions of EBN.

The enzymatically digested EBN peptides were enriched by a 3 kDa molecular weight cut-off ultracentrifugal tube (3K Omega Tubes, PALL Life Science, Port Washington, NY, USA) following the manufacturer’s protocol. Further chromatographic separation was performed on a VisionHT C18 HighLoad column (250 × 4.6 mm, 5 μm). The HPLC gradient with solvents 0.1% trifluoroacetic acid in water (A) and acetonitrile (B) was applied as 0–10 min, 5–15% (B) linear; 10–60 min, 15–30% (B) linear; 60–80 min, 30% (B) isocratic; 80–90 min, 30–5% (B) linear at a flow of 0.6 mL/min. During analysis, the column was pre-equilibrated for 20 min and set at room temperature. The peptide was detected at 214 nm. Peaks eluted at 23, 32, 35, 41, and 47 min were collected by a fraction collector. The collected peaks were dried using a centrifugal vacuum concentrator (Savant^®^ ISS110 SpeedVac^®^ concentrator, Thermo Fisher Scientific, Waltham, MA, USA) for further analysis.

### 4.3. Peptide Identification by TMT10plex nanoLC-Orbitrap Eclipse MS/MS

Prior to TMT10plex labeling, EBN digests and peptide fractions were subjected to desalting via Ziptip. Peptides were then quantified with the Pierce^®^ fluorescent quantitative assay kit (Thermo Fisher Scientific), normalized to >10 μg, and dried with SpeedVac. TMT10plex-labeled samples were then processed by desalting with Pierce^®^ C18 spin tips, followed by drying. An Easy-Spray™ PepMap™ RSLC C18 column (25 cm × 75 µm, 2 µm) was coupled to an EASY-nLC™ 1200 system for peptide separation. The nanoflow LC gradient with solvents 0.1% formic acid in water (A) and 0.1% formic acid in 80% acetonitrile (B) was applied as 0–113 min, 2–30% (B) linear; 113–119 min, 30–40% (B) linear; 119–120 min, 40–100% (B) linear; 120–130 min, 100% (B) isocratic. The eluted peptides were ionized by electrospray ionization (ESI) at +2.4 kV and transferred into the Orbitrap Eclipse™ Tribrid™ mass spectrometer (Thermo Fisher Scientific) at 325 °C (ion transfer tube temperature). Data-dependent acquisition (DDA) was performed with 1 microscan for MS1 at 60K resolution and 1 microscan for MS2 at 30 K. The full MS mass range was set to *m*/*z* 350–1500, with a first MS2 mass of *m*/*z* 110. The MS2 automatic gain control (AGC) target was 200%, the maximum injection time was 50 ms, the higher-energy collisional dissociation (HCD) energy was 38%, and the dynamic exclusion duration was 60 s. Orbitrap Eclipse MS data output was searched against the 3 Uniprot databases (downloaded on 24 September 2022): *Apodidae* (4031 entries), *C. pelagica* (3212 entries), and *Aerodramus* (319 entries), via PEAKS Studio 10.6. Peptides were generated from pepsin (pH 1.3) digestion, limited to 2 missed cleavages. Modifications included carbamidomethylation of cysteine residues (+57.02 Da) and TMT10plex (+229.16 Da) as fixed and oxidation of methionine residues (+15.99 Da) and acetylation of the protein N-terminus (+42.01 Da) as variable. The mass tolerances were set at 15.0 ppm (parent) and 0.02 Da (fragment), respectively. Peptide search results were filtered with FDR < 1% and PTM AScore or −10logP ≥ 20.

### 4.4. Targeted Peptide Quantification by LC-Triple-Quadrupole MS/MS

An Agilent ZORBAX Eclipse Plus-C18 rapid resolution HT column (2.1 mm × 50 mm, 1.8 µm, 600 bar) was coupled to an Agilent 6410 Triple Quadrupole MS/MS system for peptide separation of the EBN digest. Samples were injected at a volume of 5 µL. A 10.8 min gradient program with solvents 0.1% formic acid in water (A) and 0.1% formic acid in acetonitrile (B) was applied as 0–2 min, 5–15% (B) linear; 2–4.5 min, 15–22.5% (B) linear; 4.5–8 min, 22.5–30% (B) linear; 8–9.6 min, 30% (B) isocratic; 9.6–10.8 min, 30–5% (B) linear. EBNP3 was eluted at 1.742 min. Positive electrospray ionization (ESI+) was employed with a capillary and cone voltage of 3.5 kV and 10 V, respectively. The temperatures were set at 100 °C (ion source) and 325 °C (desolvation), respectively. Ultra-high purity nitrogen served as the nebulizing gas (10.0 L/min, 40 psi). The concentration of EBN_P3_ in the enzymatic digest was determined using the standard addition method. Specifically, aliquots of the digest were spiked with EBN_P3_ standard solutions at 4 concentrations: 500, 5000, 10,000, and 20,000 ppb, respectively. Multiple reaction monitoring was performed with a collision energy of 70 eV. The CID of [M+H]^+^ ion of EBN_P3_ (at *m*/*z* 686.4) produced product ions at *m*/*z* 86.0, 110.0, and 178.2. The most abundant product ion (at *m*/*z* 110.0) was monitored for quantification.

### 4.5. Cell Culture

HaCaT human epidermal keratinocytes (AddexBio, San Diego, CA, USA) and RAW264.7 murine macrophages (ATCC, Manassas, VA, USA) were cultured in DMEM supplemented with 10% (*v*/*v*) FBS and penicillin/streptomycin (100 U/mL and 100 μg/mL) at 37 °C in a humidified 5% CO_2_ atmosphere. For RAW264.7 cells, FBS was heat-inactivated. All reagents were from Thermo Fisher Scientific. Cell viability was determined by MTT assay (0.5 mg/mL, DMSO solubilization), with absorbance measured at 570 nm using a Multiskan™ FC Microplate Reader (Thermo Fisher Scientific).

### 4.6. DNA Transfection

The pNF-κB-Luc construct contains five NF-κB response elements (5′-GGG AAT TTC CG-3′) driving a luciferase reporter. The pFLG2-eGFP construct consists of pEGFP-N1 with the filaggrin-2 promoter controlling expression of a red-shifted eGFP. HaCaT cells were transfected with jetPRIME (Polyplus Transfection, New York, NY, USA) as described [10]. pNF-κB-Luc-transfected cells were treated with TNF-α (20 ng/mL) for 2 h together with EBN digest; dexamethasone (10 nM) served as a positive control. Cells were lysed in buffer (25 mM Tris-phosphate, pH 7.8, 2 mM DTT, 2 mM 1,2-diaminocyclohexane-N,N,N′,N′-tetraacetic acid, 10% glycerol, 1% Triton^®^ X-100). After centrifugation (16,000× *g*, 5 min, 4 °C), 75 µL of lysate was transferred to a 96-well plate, and luminescence was read on a GloMax 96 Luminometer (Promega, Madison, WI, USA).

### 4.7. Real-Time PCR Analysis

The mRNAs encoding IL-1β, IL-6, TNF-α, filaggrin, filaggrin-2, and GAPDH were quantified by real-time PCR. Total RNA was extracted from HaCaT and RAW264.7 cells using RNAzol^®^ RT reagent (Molecular Research Center, Cincinnati, IA, USA). RNA quality and quantity were assessed by NanoDrop™ (Thermo Fisher Scientific) at A260/A280 and A260/A230 ratios. Total RNA (2 μg) was treated with DNase I (New England Biolabs, Hitchin, UK) to remove contaminating genomic DNA. Then, 500 ng of RNA was reverse transcribed using PrimeScript™ RT Reagent Kit (TaKaRa, Kusatsu, Japan). Real-time PCR was performed on a LightCycler^®^ 480 System (Roche, Basel, Switzerland) with primer sequences as follows:

Human primers: IL-1β (sense 5′-ATG GCA GAA GTA CCT AAG CTC GC-3′, antisense 5′-ACA CAA ATT GCA TGG TGA AGT CAG TT-3′); IL-6 (sense 5′-GAG AGT AGT GAG GAA CAA GCC AGA GC-3′, antisense 5′-CTA CAT TTG CCG AAG AGC CCT CAG G-3′); TNF-α (sense 5′-ATG AGC ACT GAA AGC ATG ATC CGG-3′, antisense 5′-GCA ATG ATC CCA AAG TAG ACC TGC CC-3′); filaggrin (sense 5′-GCT GAA GGA ACT TCT GGA AAA GG-3′, antisense 5′-GTT GTG GTC TAT ATC CAA GTG ATC-3′); filaggrin-2 (sense 5′-CTG TGG TCA TTC ATG GAG TGG-3′, antisense 5′-CCC TAG AAG GGC TAA TGT GTG A-3′); GAPDH (sense 5′-ACA ACT TTG GTA TCG TGG AAG G-3′, antisense 5′-GCC ATC ACG CCA CAG TTT C-3′). Mouse primers: IL-1β (sense 5′-GTG GTA TTC TCC ATG AGC TT-3′, antisense 5′-TTC ATC ACA CAG GAC AGG TA-3′); TNF-α (sense 5′-AGT GAC AAG CCT GTA GCC-3′, antisense 5′-AGG TTG ACT TTC TCC TGG-3′); GAPDH (sense 5′-AAC GGA TTT GGC CGT ATT GG-3′, antisense 5′-CTT CCC GTT CAG CTC TGG G-3′). Amplification was performed for 45 cycles (95 °C for 30 s, 55 °C for 30 s, 72 °C for 20 s). Relative mRNA levels were determined by calculating 2^−ΔΔCt^ values.

### 4.8. Measurement of ROS

HaCaT keratinocytes were seeded at 5 × 10^4^ cells/well in 96-well optical-bottom microplates (for plate reader) or onto sterile coverslips (for confocal microscopy). After 24 h, cells were pre-incubated with or without EBN peptide for 2 h; positive control cells received 20 mM N-acetyl-L-cysteine (NAC) for 1 h. Cells were then stimulated with TNF-α for 5 min. ROS levels were detected by incubating 10 µM DCFH-DA for 20 min. For confocal imaging, cells on coverslips were fixed with 4% PFA for 10 min at room temperature, mounted with DAPI-containing medium, and imaged using a Leica SP8 confocal microscope. For quantitative analysis, DCF fluorescence in separate wells was measured kinetically over 1 h using a FlexStation^®^ reader (λ_ex_/λ_em_ 492/527 nm, Molecular Devices Sunnyvale, CA, USA). Fluorescence intensities were normalized to protein concentration determined by Bradford protein assay.

### 4.9. Western Blot Analysis

HaCaT keratinocytes were seeded at 3 × 10^5^ cells/well in 6-well plates (for total protein) or 12-well plates (for phosphorylation). After 24 h, cells were treated with TNF-α and/or EBN_P3_. For total protein analysis, cells were lysed in buffer containing 150 mM NaCl, 10 mM HEPES, 1 mM EDTA, 1 mM EGTA, 1% NP-40, 0.01% SDS, 0.1 M Tris-HCl (pH 7.6), and protease inhibitors (aprotinin, leupeptin, benzamidine, pepstatin A, each at 1:1000 except benzamidine 1:200). For phosphorylation analysis, cells were lysed directly in direct lysis buffer (0.125 M Tris-HCl pH 6.8, 4% SDS, 20% glycerol, 2% 2-mercaptoethanol, 0.02% bromophenol blue). All lysates were denatured at 95 °C for 15 min with vortexing every 5 min. Proteins were separated by 10% SDS-PAGE (60–85 V, room temperature), transferred to nitrocellulose membranes (4 °C), and blocked with 5% non-fat milk or BSA in TBST (pH 7.6) for 1 h. Primary antibody incubation was performed overnight at 4 °C, followed by HRP-conjugated secondary antibody (1:2000) for 2 h at room temperature. Bands were visualized by ECL and quantified using a ChemiDoc imaging system. Relative protein expression was normalized to α-tubulin.

### 4.10. Immunofluorescence Staining

HaCaT keratinocytes were seeded at 5 × 10^4^ cells per well onto sterile coverslips. Cultured keratinocytes and ex vivo mouse dorsal skin sections were fixed with 4% paraformaldehyde (PFA) for 10 min and 30 min, respectively. Samples were then blocked for 2 h at room temperature in PBS containing 5% BSA, with or without 0.1% Triton X-100 for permeabilization as appropriate. After blocking, samples were incubated overnight at 4 °C with anti-filaggrin primary antibody (1:50). Following primary antibody incubation, samples were incubated with Alexa Fluor 647-conjugated secondary antibody (1:200; Cell Signaling Technology, Danvers, MA, USA) for 2 h at room temperature in the dark. Samples were mounted on glass slides using mounting medium containing DAPI and imaged using a Leica SP8 confocal microscope with a 63× oil immersion objective.

### 4.11. Induction of Dermatitis-like Skin Lesion in Mice

C57BL/6 mice were supplied by the Animal and Plant Care Facility at The Hong Kong University of Science and Technology (HKUST). Experimental procedures were approved by the university’s Animal Ethics Committee (Reference No.: (20-104) in DH/HT&A/8/2/2 Pt. 2). Mice were housed at constant temperature and humidity under a 12 h light/dark cycle with free access to food and water. Eight- to twelve-week-old mice were randomly assigned to four groups (*n* = 4): control (vaseline), DNCB (7% *w*/*w*), dexamethasone (0.12% *w*/*w*), and EBN_P3_ (0.01, 0.1, or 1 mg/mL). Before the experiment, mice were anesthetized with isoflurane and shaved. Except for controls, dorsal skin was sensitized with 7% DNCB cream on days 1 and 6. Thereafter, daily applications of the respective treatment creams (dexamethasone or EBN_P3_) were administered for 10 days. Mice were euthanized by CO_2_ inhalation at the end of the experiment.

### 4.12. Skin Section Staining

After CO_2_ euthanasia, dorsal skin was isolated, rinsed with cold PBS, fixed in 4% PFA for 2 h at room temperature, and dehydrated overnight at 4 °C in 30% sucrose/PBS. The skin was embedded in OCT, frozen at −80 °C overnight, and sectioned at 10 μm (−20 °C) using a CryoStar™ NX70 cryostat (Thermo Fisher Scientific). For H&E staining, a commercial kit was used; sections were mounted with DPX and imaged with a Zeiss Axio inverted phase microscope (10× objective, Jena, Germany). For toluidine blue staining, a 0.1% solution in 1% NaCl (pH 2.3) was prepared. Sections were pre-wet with PBS, washed twice for 5 min, dried, stained for 5–30 min, rinsed with water, differentiated with 0.5% glacial acetic acid, dehydrated through 95% and absolute ethanol, mounted with DPX, and imaged similarly.

### 4.13. ELISA

Isolated mouse dorsal skin (30–50 mg) was placed into microtubes. Total protein was extracted with 200 µL of RIPA buffer (150 mM NaCl, 5 mM EDTA, 50 mM Tris-HCl, pH 8.0, 1% *w*/*v* NP-40, 0.5% *w*/*v* sodium deoxycholate, 0.1% *w*/*v* SDS) containing protease inhibitors (aprotinin, leupeptin, benzamidine, pepstatin A). Samples were sonicated on ice for 5 cycles (30 s pulses at 50% amplitude, 5 s rest between cycles). IL-6, IL-10, and FLG levels in the lysates were measured by ELISA following the manufacturer’s protocols, with absorbance read at 450 nm (signal) and 570 nm (background) using a Multiskan™ FC Microplate Reader (Thermo Fisher Scientific).

### 4.14. Statistical Analysis

Data from at least three independent experiments are expressed as mean ± SEM. Statistical comparisons were performed using one-way ANOVA with Dunnett’s post hoc test or two-way ANOVA with Tukey’s post hoc test, as appropriate, using GraphPad Prism 8.3.0. Significance levels are indicated as * *p* < 0.05, ** *p* < 0.01, and *** *p* < 0.001.

## Data Availability

The original contributions presented in this study are included in the article/Supplementary Material. Further inquiries can be directed to the corresponding author.

## Supplementary Materials

The following supporting information can be downloaded at: https://www.mdpi.com/article/10.3390/ph19040649/s1. Figure S1: Bioactivity, binding prediction, and quantification of EBN_P3_. (A) HaCaT cell viability. (B) Mouse dorsal skin thickness. (C) Predicted TNF-α–EBN_P3_ binding model. (D) LC–MS/MS quantification of EBN_P3_ in EBN digests.
