# Supplementary material for: Short Peptide with Sequence of LAGAAHF, Identified from Edible Bird’s Nest, Reduces Dermatitis Symptoms in Mice
Source: Pharmaceuticals (Basel). 2026 Apr 21;19(4):649. doi: 10.3390/ph19040649 (PMC13119178; doi:10.3390/ph19040649)
Supplement: Supplementary file 1 [file pharmaceuticals-19-00649-s001.zip › pharmaceuticals-4227864-supplementary.pdf]

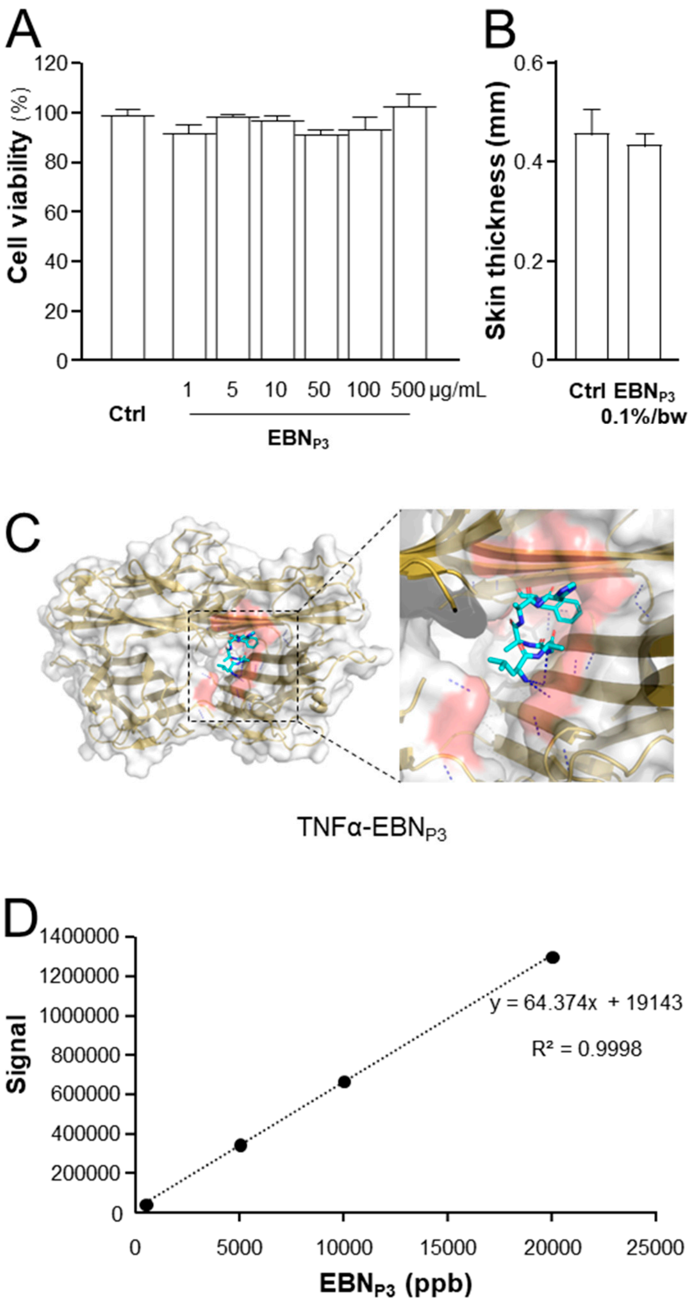

Figure S1. Bioactivity, binding prediction, and quantification of EBNP3.(A) HaCaT cell viability. HaCaT cell viability assessed after 48 hours incubations of 1, 5, 10, 50, 100, and 500 µg/mL of EBNP3. A statistical difference comparing with the control group was analyzed by one-way ANOVA, and presented herein as mean ± SEM, $n = 3$. **(B)** Mouse dorsal skin thickness. Skin thickness measured 2 weeks after topical application of 1,000 mg/kg EBNP3 on mouse dorsal skin. A statistical difference comparing with the control group was analyzed by one-way ANOVA, and presented herein as mean ± SEM, $n = 3$. **(C)** Predicted TNF-α–EBNP3 binding model. The X-ray crystal structure of TNF-α (PDB ID: 2AZ5, https://www. rcsb.org/), a key pro-inflammatory cytokine in the NF-κB signaling pathway and the pathology of skin psoriasis, was selected as the target. Peptide-protein docking was employed to explore the binding affinity between EBNP3 and TNF-α [20], with potential inhibitory effects and therapeutic applications for skin inflammation. Alphafold2 colab (https://colab.research.google.com/github/sokrypton/ColabFold/blob/main/AlphaFold2.ipynb) was used to predict

five highly ranked structures (pLDDT > 60%) for EBN_{P3} [16]. The active sites of TNF-α were predicted using the CASTp server (http://sts.bioe.uic.edu/castp/index.html?2pk9), which measured and characterized the cavities, pockets, and channels of the TNF-α protein structure, and predicted the key amino acids binding to potential inhibitors [30]. The solvent-accessible surface area and volume of the TNF-α pocket were predicted to be 2,743.256 $Å^2$ and 4,514.068 $Å^3$ by CASTp. The HDOCK server (http://hdock.phys.hust.edu.cn/) was used to carry out the docking analysis between EBN_{P3} and TNF-α, with Certolizumab Fab (PDB ID: 5WUV) serving as the positive control. The interactions between polar atoms in the best docked complex were visualized by PyMOL™ 2.5.5, with potential contact areas on TNF-α filled in red and molecular interactions indicated by blue dashed lines. The potential binding interactions between EBN_{P3} and TNF-α were predicted at the Gln61, Tyr119, Leu120, Gly121, Gln149, and Tyr151 active site residues on TNF-α chain A; the Leu57, Tyr59, Ser60, Gln61, Tyr119, Leu120, Gly121, Gly122, and Tyr151 active site residues and Ile58 inactive site residue on TNF-α chain B; the Lys11 active site residue on TNF-α chain C; and the Leu55 and Leu157 active site residues on TNF-α chain D. The most prominent docking score and shortest ligand rmsd values for EBN_{P3} were -203.58 and 87.22 Å, respectively, compared to -256.90 and 86.34 Å for the certolizumab light domain, a commercial TNF-α monoclonal antibody. **(D)** LC-MS/MS quantification of EBN_{P3} in EBN digests. Standard addition calibration curve for EBN_{P3} determined by LC-MS/MS. The curve was constructed by spiking an EBN enzymatic digest with EBN_{P3} standard at concentrations of 500, 5,000, 10,000, and 20,000 ppb, respectively.

Disclaimer/Publisher's Note: The statements, opinions and data contained in all publications are solely those of the individual author(s) and contributor(s) and not of MDPI and/or the editor(s). MDPI and/or the editor(s) disclaim responsibility for any injury to people or property resulting from any ideas, methods, instructions or products referred to in the content.

https://doi.org/10.3390/ph19040649
